# Identification and applications of disease-associated differential human and bacterial proteins with metaproteomic evidence

**DOI:** 10.1007/s13755-025-00369-z

**Published:** 2025-08-29

**Authors:** Jamie Canderan, Moses Stamboulian, Yuzhen Ye

**Affiliations:** Luddy School of Informatics, Computing, and Engineering, Indiana University, Bloomington, IN 47408 USA

## Abstract

The gut microbiome plays a fundamental role in human health and disease. Individual variations in the microbiome and the corresponding functional implications are key considerations to enhance precision health and medicine. Metaproteomics has recently revealed protein expression that might be associated with human health and disease. Existing studies focused on either human proteins or bacterial proteins that can be identified from (meta)proteomics data sets, but not both. In this study, we examined the feasibility of identifying both human and bacterial proteins that are differentially expressed between healthy and diseased individuals from metaproteomics data sets. We further evaluated different strategies of using identified peptides and proteins for building predictive models. By leveraging existing metaproteomics data sets and a tool that we have developed for metaproteomics data analysis (MetaProD), we were able to derive both human and bacterial differentially expressed proteins that could serve as potential biomarkers for all diseases we studied. We also built predictive models using identified peptides and proteins as features for prediction of human diseases. Our results showed peptide-based identifications over protein-based ones often produce the most accurate models and that feature selection can offer improvements. Prediction accuracy could be further improved, in some cases, by including bacterial identifications, but missing data in bacterial identifications remains problematic.

## Introduction

The gut microbiome has been shown to play a fundamental role in human health and disease [1, 2] and gut dysbiosis has been reported to be associated with many diseases, such as Crohn’s disease (CD) and ulcerative colitis (UC) [3]. Precision medicine efforts focus on providing targeted and efficient treatments toward individuals [4], but gut microbial composition can vary significantly between individuals or within the same individual across time [5–7]. Identifying bacterial species or protein composition within an individual may prove relevant to providing effective treatment and highlights the importance of computational proteomics to consider bacterial identification and expression.

Genomics-based techniques are often used for precision medicine given the amount of available data and known associations with diseases, such as cancer [8] and type II diabetes [9]. Genomics studies may fail to capture important information, such as protein abundance, post-translation modifications, enzyme activity, or protein degradation [10, 11]. This information, however, might be captured by proteomics-based methods.

Proteomics-based approaches have shown importance in biomarker and drug discovery research because factors, such as age, disease, and gender, could affect protein expression and activity [12]. Proteomics may also aid in biomarker identification for heterogeneous diseases, such as type 2 diabetes [13]. Mass spectrometry (MS) based techniques are frequently used in proteomics studies and MS has been used in such cases as mapping the human proteome [14] and identifying bacterial function [15] and composition [16]. MS approaches have helped identify changes in response to disease [17] and been used to identify bacterial proteins to inform treatments based on these proteins [18].

Previous work has paired MS data with machine learning approaches for disease classification in chronic kidney disease [19], inflammatory bowel disease (IBD) [20], or Alzheimer’s disease [21] amongst others. But most analysis tends to focus on human proteins only. The importance of bacterial species and protein expression on disease and treatment emphasizes the need to enhance existing methods and develop new ones that factor in bacterial expression. For some diseases, such as IBD, it has been suggested that new biomarkers are needed to enhance accuracy of diagnostics and more accurately reflect disease activity [22]. The identification of bacteria-based biomarkers could aid in this endeavor, especially as has been shown that the microbiome remains a target for therapy [23].

We previously developed MetaProD [24] for fast and efficient identification of peptides and proteins from MS/MS spectra. MetaProD was benchmarked using mock and real metaproteomic data sets and compared to some other similar pipelines that involve identifying bacteria from MS data. MetaProD has the ability to impute missing data with PEMM [25] and uses DEqMS [26] to identify differentially expressed proteins. PEMM is a penalized expectation- maximization algorithm designed to impute missing values in MS data selected based on previous work evaluating imputation strategies in mass spectrometry [27]. DEqMS is a tool to identify differentially expressed proteins and works similarly to Limma [28] but with consideration to how many peptide spectral matches (PSMs) were identified per protein. MetaProD utilizes a two-step search with the first step searching against high-abundance proteins to profile the species composition and the second step searching against the full proteome of identified species to achieve speedup. It also supports searching using multiple search engines to improve results.

Here, we enhanced the MetaProD [24] metaproteomics pipeline with the ability to calculate peak areas for quantitation. We used these peak areas to determine human and bacterial expression levels in label-free MS data for different disease phenotypes (Crohn’s disease, ulcerative colitis, type-1 diabetes (T1D), and two acute leukemia (AL) disease phenotypes). We identified differentially expressed human and bacterial proteins that could be used as biomarkers in precision medicine for each disease. Finally, we evaluated peptide and protein expression data to determine whether identification of bacterial peptides and proteins can enhance the accuracy of machine learning (ML) models and compared the performance of using peptide peak area features versus protein.

## Methods

### Curation of metaproteomics MS data sets

Human gut metaproteomics data sets were gathered from four separate studies. Two data sets containing samples from patients with Crohn’s disease or ulcerative colitis along with healthy controls (C) were gathered from two studies identified as CD2 [29] (containing children) and CD3 [30] (containing adults). A data set identified as T1D1 [31] containing samples from patients diagnosed with seropositive (SP) type-1 diabetes along with healthy controls (C) was obtained and these SP samples were labelled as the T1D phenotype. A final data set identified as AL1 [32] containing samples from patients with acute myeloid leukemia (AML) and acute lymphoblastic leukemia (ALL) was also included. The summary of the data sets used, the number of patients, and the number of files analyzed is available in Table 1. All files available in the original data sets were analyzed except in the case of the CD3 data set where 452 out of 457 files were analyzed due to processing errors with five of the files. Methods for data collection, sample processing, and MS spectra generation are included in the original studies.

**Table 1:** Summary of data sets used and breakdown by phenotype

Data Set	# Patients	# Files
CD2	174 (61 CD, 52 UC, 61 C)	174
CD3	89 (39 CD, 26 UC, 24 C)	452
T1D1	32 (17 SP, 15 C)	32
AL1	56 (47 AML, 9 ALL)	421

### Mass spectrometry analysis with MetaProD

The metaproteomic data sets were analyzed using a modified MetaProD pipeline that included the addition of peak area as a method for peptide quantitation. MetaProD was run with settings of 10 parts-per-million precursor and fragment tolerance, trypsin as the protease with specific digestion, carbamidomethylation of cysteine as a fixed modification, and oxidation of methionine as a variable modification to correspond to the methods used in the original studies. MetaProD uses a two-step search. The first step involved using a single search engine (Comet [33]) combined with a FASTA protein database containing only highly-abundant bacterial proteins from bacterial reference and pan-proteomes downloaded from UniProt [34] (release 2023_04) to quickly identify bacterial proteomes present in the sample. The proteins belonging to each proteome were totalled by normalized spectrum abundance factor [35] (NSAF) and the top 90% of proteomes by NSAF were selected for the second step. The second step included a multi-engine search (MSGF + [36], Comet, X!Tandem [37], and OMSSA [38] for this paper) and a FASTA file containing all proteins for identified proteomes. Both steps also included the full human proteome downloaded from UniProt and proteins listed in the Common Repository of Adventitious Proteins [39]. The results from the multi-engine search were used to generate a final list of identifications using PeptideShaker [40]. These results were imported into an SQL database and species, PSM, peptide, or protein reports were exported for use in other programs.

The data sets used in the study were label-free and MetaProD was originally designed to work with multiplexed data so the original MetaProD pipeline was expanded to use MZMine3 [41] to quantify peptide peak areas by using the search results from the previous step and the targeted peak detection function. The targeted peak detection allowed for the isolation of specific peptide peaks based on the results from PeptideShaker.

### Identification of differentially expressed proteins

Each data set was filtered to only include peptides found in at least 50% of the samples for that study corresponding to the methodology used in the original MetaProD paper. The peptides were then separated by phenotype, the peak areas were *log*_2_ transformed, median centered, and any missing peak areas were imputed using the PEMM package in R using the default settings. Peptides were grouped by protein accession and median centered to generate protein expression data. A pseudo-count of one was added to all PSM values so any peptide (including the imputed ones) was considered to have at least a single PSM prior to running DEqMS. DEqMS was then used on each individual data set to find differentially expressed proteins using the default settings.

The protein expression from the CD2, CD3, and T1D1 data sets were compared to healthy controls from the respective data set. The CD data sets compared both the CD and UC phenotypes to healthy controls and the T1D data set compared the T1D phenotype to healthy controls. The AL data set did not contain healthy controls so the two leukemia phenotypes (AML and ALL) were compared to each other.

### Predictive models for human disease prediction

The peak area abundances for the peptides were divided into human and bacterial peptides (labeled as “human” and “bacterial”) based on the previously inferred protein assignment as part of the MetaProD pipeline. Human and bacterial peptides were also joined to form a group labeled as “combined”. These peptides were filtered to require expression in at least 75% of samples (60% for CD2 due to fewer proteins meeting the 75% threshold in this data set).

**Fig. 1:**
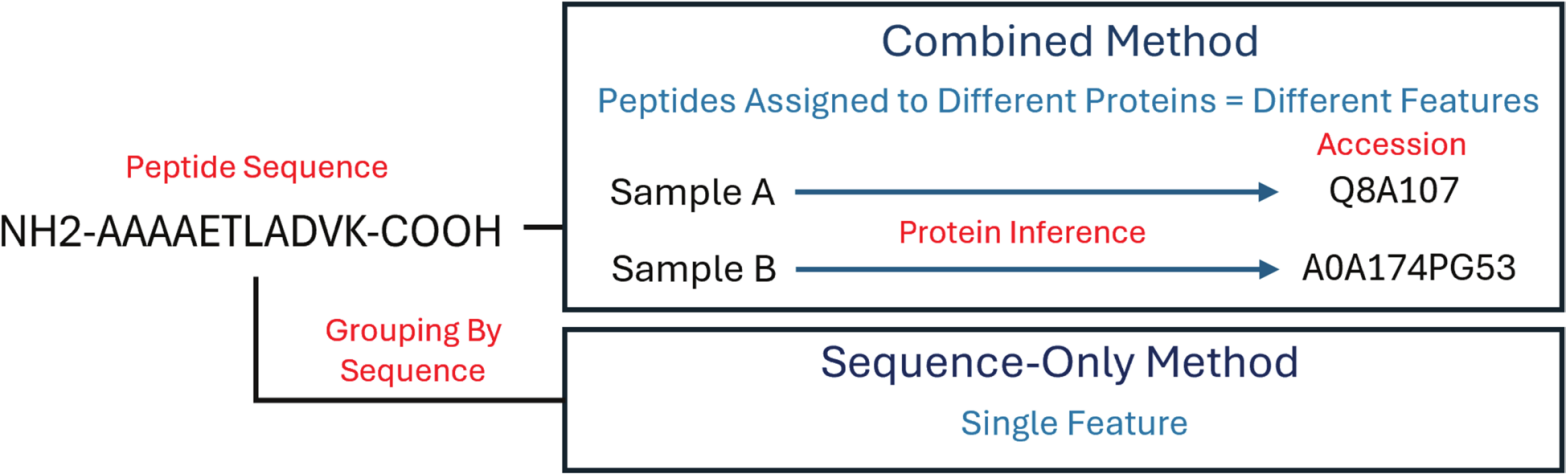
Difference between the “combined” and “sequence-only” methods of assigning peptide features in MetaProD

Another category labeled as “sequence-only” was created that consisted of peptides grouped solely by modified sequence (which factors in any post-translation modifications) regardless of inferred protein assignment by MetaProD as described in Fig. 1 to evaluate the possible effects of protein inference on classification. A singular peptide sequence may be inferred to belong to different proteins depending on the sample because protein inference in MetaProD is based on individual samples. For the “human”, “bacterial”, and “combined” categories, a peptide peak area feature is therefore based on both the sequence and the protein inference and for the “sequence-only” category, this feature is only based on sequence. The “sequence-only” group was also filtered as described above to require expression in a certain percentage of samples.

Protein peak areas were also generated by using peak area abundances for peptides (post-filtering). Peptides were grouped by accession and protein expression was determined by the median of the peak areas of the peptides assigned to the protein. Proteins were also divided into “human” and “bacterial” categories after filtering and a “combined” group containing all proteins from both.

Peptide and protein abundances based on peak areas were used in a Python Scikit-learn [42] pipeline to build predictive models for phenotype prediction. Performance of the predictive models was evaluated using the receiver operating character (ROC) curves along with area under the curve (AUC) values using the repeated stratified k-fold cross validation (5 folds, 10 repeats) feature in Scikit-learn. A different partition was used for each repeat. For the CD2 and CD3 data sets, multiple classes are present (CD, UC, and C), and AUC scores are based on one-versus-rest (OvR) micro-averaging where true and false positive rates from each class are aggregated together to form the final metric. Missing values were replaced with a constant value zero to avoid the potential for data leakage that might occur when using PEMM across an entire data set. Values were standardized by removing the feature (peptide/protein) median and scaled to the quantile range. Recursive feature elimination with cross validation using random forest (RF-RFECV) with a step size of 0.05, linear support vector classification with an L2 penalty (Ridge), and select K-best using ANOVA F-value and 10 features (K-Best) were evaluated as a feature selection step in the pipeline using the relevant Scikit-learn packages versus using no feature selection (None). Model classification was performed using random forest (RF) [43], support vector machines (SVM) [44], and k-nearest neighbors (KNN) [45] using the default settings for all combinations.

## Results

### Summary of the metaproteomic identification results

A total of 4,790,211 non-unique peptide spectrum matches for the CD2 data set (2,147,672 human and 2,642,539 bacterial), 2,827,060 for the CD3 data set (984,484 human and 1,842,576 bacterial), 403,379 for the T1D1 data set (84,442 human, and 318,937 bacterial), and 3,677,302 for the AL1 data set (1,303,123 human, and 2,374,179 bacterial) were identified during the initial MetaProD pipeline. More bacterial identifications were found in each data set with the CD3, T1D1, and AL1 data sets showing significantly more bacterial identifications compared to human.

Table 2 indicates the number of peptides (unique PSMs) identified before sample percent filtering and the total and percents remaining after filtering for the “human”, “bacterial”, and “combined” subsets. Significantly higher amounts of bacterial peptides and proteins were filtered compared to human and this led to fewer remaining bacterial peptides to use as features compared to human despite significantly higher amounts of initial identifications. This similarly results in far fewer bacterial proteins remaining once peptides are grouped into proteins for all data sets and illustrates a challenge in balancing data loss versus imputation when including bacterial proteins. Bacterial features also had significantly higher amounts of missing data (values needing imputation) post filtering for both peptides and proteins in all data sets.

**Table 2:** Breakdown of the # of peptide features pre and post filtering and the resulting # of proteins (% missing values in brackets)

Data Set	Subset	# Pep. Pre	# Pep. Post	# Prot. Post
CD2 (children)	Human	72,380 (94.42)	600 (26.16)	166 (17.33)
	Bacterial	286,403 (97.79)	42 (36.14)	27 (32.76)
CD3 (adults)	Human	29,738 (90.14)	692 (13.14)	151 (7.50)
	Bacterial	303,005 (96.64)	96 (20.20)	43 (17.59)
T1D1	Human	13,638 (91.09)	166 (16.95)	47 (9.27)
	Bacterial	191,267 (97.01)	32 (22.59)	17 (18.93)
AL1	Human	34,152 (84.82)	1905 (10.41)	273 (6.84)
	Bacterial	459,209 (95.59)	59 (18.43)	23 (14.67)

**Fig. 2:**
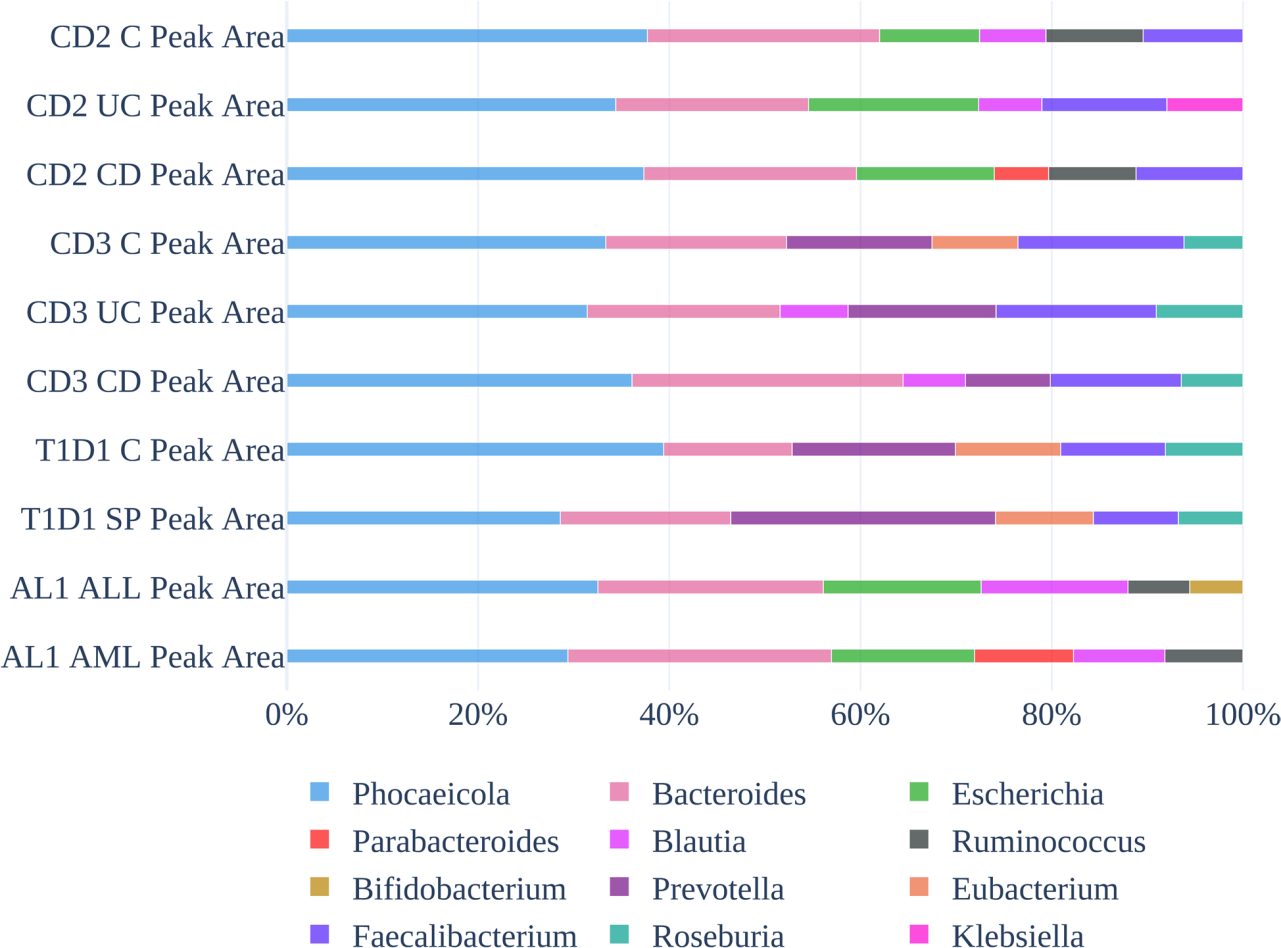
Top-6 genus breakdown showing genus differences between the T1D1, AL2, CD2, and CD3 data sets based on peak area sums for all peptides

The genus-level composition of the top 6 non-human genera for the CD2/CD3 (for the CD, UC, and control phenotypes), T1D1 (T1D and C phenotypes), and AL1 (AML and ALL phenotypes) data sets using peak area sum is reflected in Fig. 2. The phenotypes within each data set had the *Phocaeicola* genus as the most expressed with the *Bacteroides* genus also highly expressed for all but the genus expression diverged after this point. Even within the same data set, there tended to be notable differences in genera between phenotypes, which could potentially be attributed to disease-based changes. For the CD2 data set, for example, *Parabacteroides* was only found in the top-6 gen-era for the CD phenotype (10th for UC and 8th for C), *Klebsiella* was only found in the UC phenotype (21st for CD and 12th for C), and CD was lacking *Blautia* in the top 6 (9th) compared to C and UC. *Parabacteroides* was found in the top 6 in the AL1 data set for the AML phenotype but was 13th for the ALL phenotype. *Bifidobacterium* was in the top-6 for the ALL phenotype, but 11th for the AML phenotype.

### Identification of differentially expressed proteins associated with different diseases

**Fig. 3:**
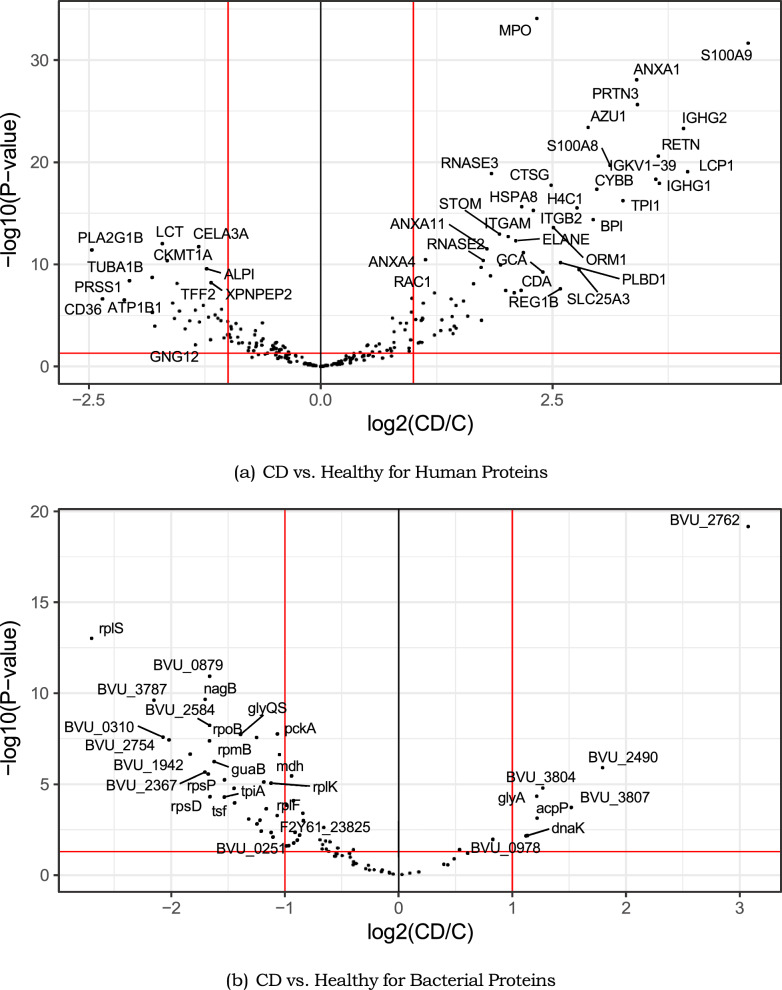
Volcano plots from the CD2 data set showing potential differentially expressed human and bacterial proteins with the red lines indicating cutoffs of a p-value of 0.05 (y-axis) and absolute FC of 2 (x-axis)

Both human and bacterial differentially expressed proteins/genes were derived from the metaproteomic data sets. Volcano plots were generated for the CD2 data set (CD/C) data set to show the differentially expressed human and bacterial genes generated by the MetaProD pipeline with a p-value of < 0.05 and an absolute-value fold-change (FC) of ± 2. Figure 3 shows the differentially expressed human genes in contrast to the differentially expressed bacterial genes (the red lines indicate the p-value and fold-change cutoffs with the upper left and upper right indicating proteins considered to be differentially expressed). For the human genes in Fig. 3a, there was a noticeable increase in genes more expressed in the CD phenotype, but for the bacterial genes in Fig. 3b, there was a noticeable increase in the genes more expressed in the healthy phenotype.

The CD2 data set (CD/C) showed a total of 130 possible differentially expressed genes (91 human, 39 bacterial), the CD3 data set (CD/C) showed 20 (9 human, 11 bacterial), the T1D1 data set (T1D/C) showed 3 (3 human), and the AL1 data set (ALL/AML) showed 15 (6 human, 9 bacterial). The CD3 and AL1 data sets had more differentially expressed bacterial genes compared to human but the T1D1 data set did not have any identified differentially expressed bacterial genes. Table 3 shows the top-two (top positive and top negative) differentially expressed genes identified by the MetaProD pipeline (the T1D1 data set did not have a differentially expressed gene with a positive FC). For CD2 and CD3, the human genes of S100A9 (calcium binding protein A9) [46] and IGHG1 (immunoglobulin heavy constant gamma 1) [47] have previously identified as genes of interest as is the case with REG1A (pancreatic stone protein) for type-1 diabetes [48]. *Phocaeicola vulgatus* (formerly *Bacteroides vulgatus*) had 3 of the top differentially expressed genes and this species is known to be associated with the gut microbiome [49]. The *P. vulgatus* gene rplS is the large ribosomal subunit protein bL19, the gene purC is a phosphoribosylaminoimidazolesuccinocarboxamide synthase, which has been studied in *E. coli* [50] and the gene rny is ribonuclease Y, which has been studied in *B. subtilis* [51]. *R. gnavus* is also known to be associated with the gut microbiome [52] and the gene HMPREF1201 01274 is associated with the protein purine nucleoside phosphorylase, which has previously be studied in *E. coli* [53].

**Table 3:** Top two differentially expressed genes (one with positive *log*_2_FC and one with negative *log*_2_FC) for each data set

Data set	Phenotype	Species	Gene	*log*_2_FC
CD2	CD/C	Human	S100A9	4.60
		*P. vulgatus*	rplS	− 2.69
CD3	CD/C	Human	IGHG1	1.52
		*P. vulgatus*	purC	− 1.47
T1D1	T1D/C	Human	REG1A	− 1.84
ALL	ALL/AML	*P. vulgatus*	rny	2.45
		*R. gnavus*	HMPREF1201 01274	− 1.15

### Predictive models for human disease prediction

**Fig. 4:**
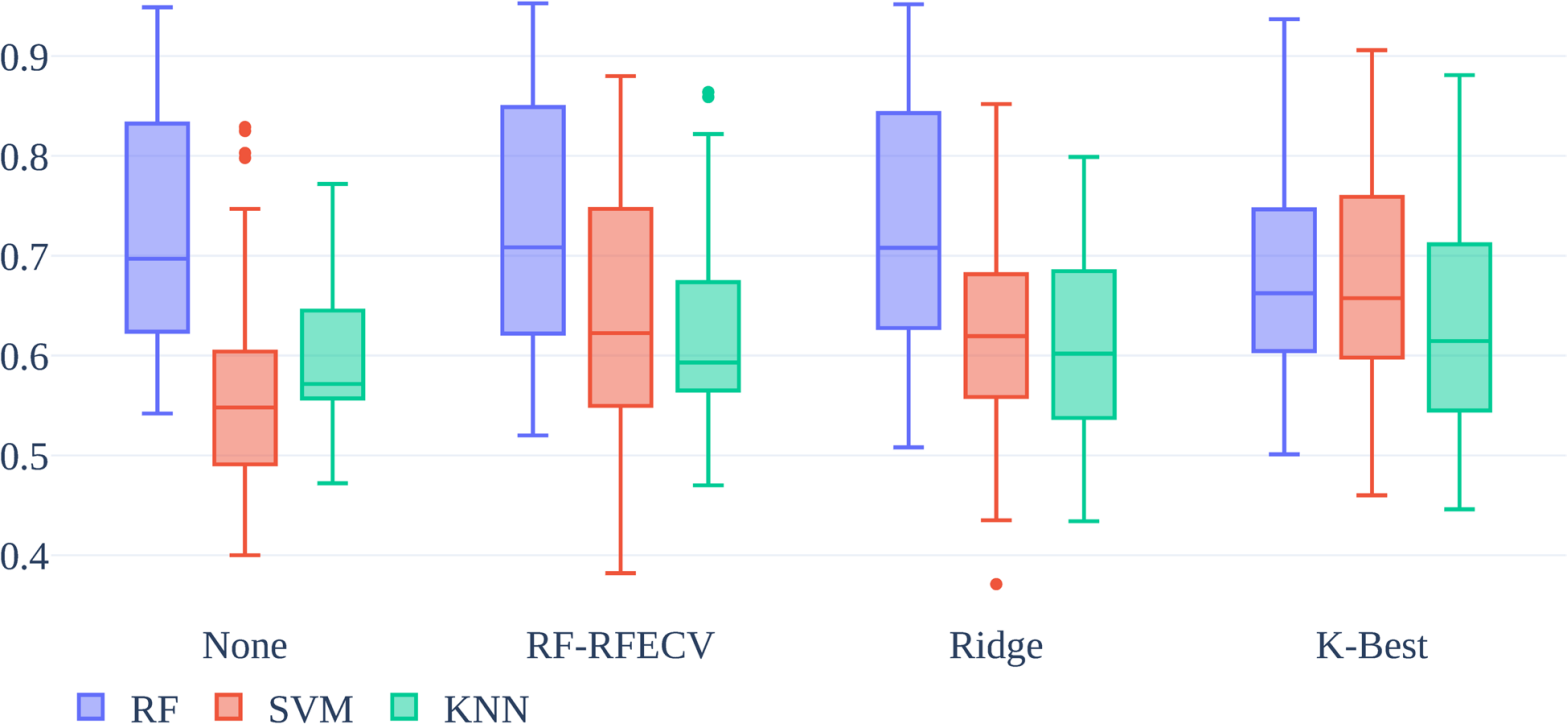
Boxplot of AUC scores showing median and range across all data sets for the 3 model types (RF, SVM, and KNN) without feature selection (None) and with RF-RFECV, Ridge, K-best feature selection

Figure 4 illustrates boxplots of the AUC score for each model used (RF, SVM, and KNN) both with and without feature selection. Each group in each data set showed that RF produced the highest median AUC scores with an overall median across all data sets and groups of 0.697 for None, 0.709 for RF-RFECV, 0.708 for Ridge, and 0.663 for K-Best versus 0.548, 0.623, 0.620, and 0.658 for SVM and 0.572, 0.593, 0.602, and 0.615 for KNN. Feature selection offered small improvements for all classifiers, with mean runtimes of 5.53 s per group for no feature selection and 397 s per group with feature selection. Based on the overall improved performance, RF using RF-RFECV was selected as the model for further analysis.

**Fig. 5:**
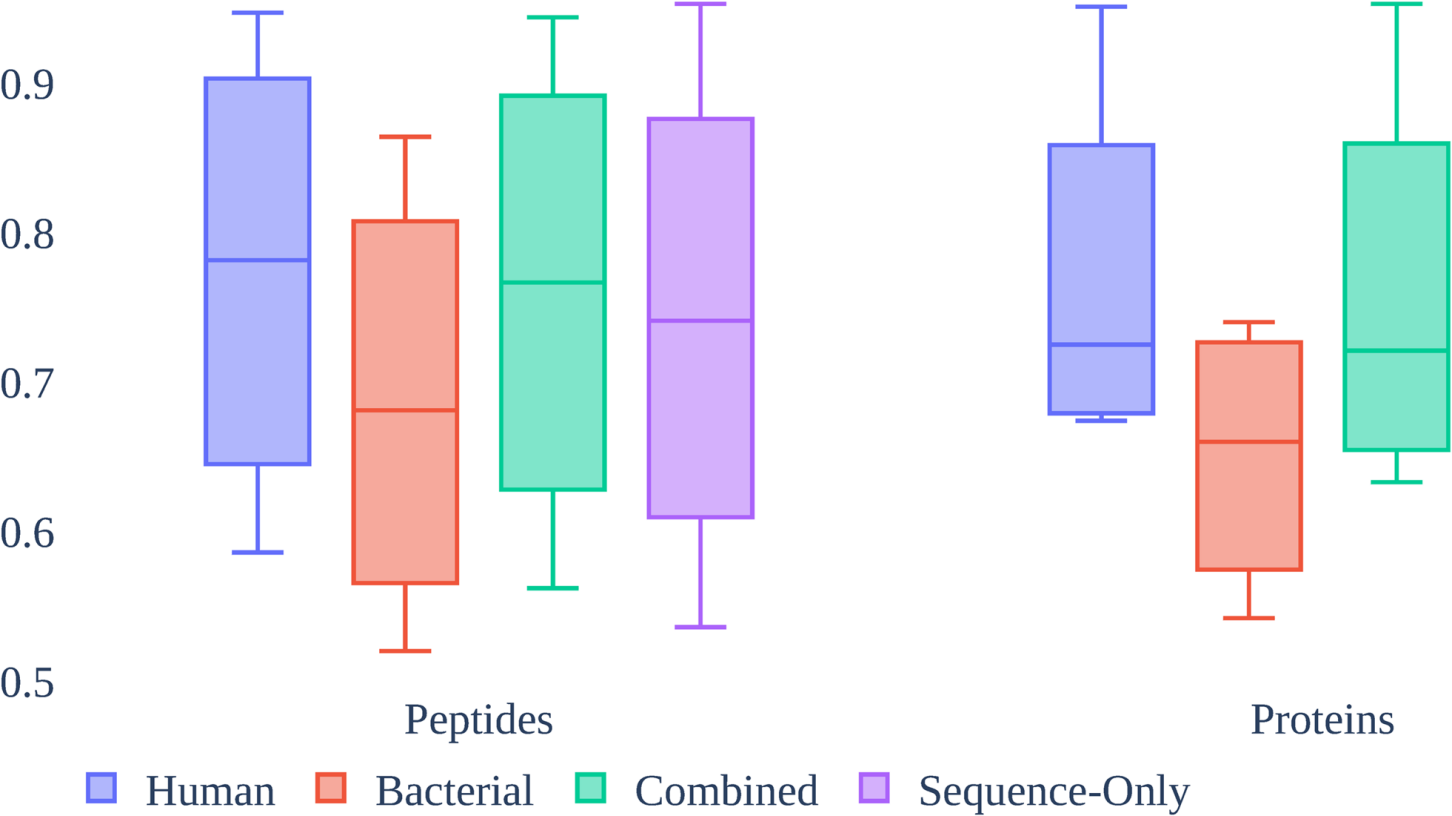
Boxplot of AUC scores across all data sets for the RF model with RF-RFECV feature selection comparing using peptide vs. protein data

Figure 5 shows a boxplot reflecting the AUC scores for the RF model with RF-RFECV feature selection for each data set and phenotypes comparing using peptide data versus protein data for each group. The “human” subgroup performance in both peptides and proteins was similar to that of “combined” with medians of 0.782 versus 0.767 in peptides and 0.725 versus 0.721 in proteins. Both groups showed that “bacterial” performance alone was lower than other groups with medians of 0.681 and 0.660 for peptides and proteins. The “sequence-only” subgroup also showed lower performance than the “combined” group for peptides. Every group showed increased performance when using peptide-data only.

Table 4 shows the mean AUC scores generated from the ROC curves for the RF classifier with RF-RFECV feature selection broken down by data set, phenotype, and subset. The higher AUC score achieved by either the peptides based model or proteins based model is bolded for each setting. Human proteins out-performed human peptides except with the AL1 data set, but most other cases (bacterial or combined) saw an increased performance with peptide data. For the peptide data, the “combined” groups tended to perform similarly or better than the other groups in the peptide data with a similar AUC in the CD2 and AL1 data sets, a higher AUC in the T1D1 data set, but a lower AUC in the CD3 data set. For proteins, the human group was generally the best with only the CD2 data set showing a similar performance to “combined”.

**Table 4:** Mean AUC score of the RF model using RF-RFECV built using different subsets of peptides and proteins identified from each data set

Data Set	Phenotype	Subset	Peptides	Proteins
		Human	0.875	**0.895**
CD2 (children)	CD vs. UC vs. C	Bacterial	**0.712**	0.695
		Combined	0.875	**0.899**
		Human	0.632	**0.647**
CD3 (adults)	CD vs. UC vs. C	Bacterial	**0.587**	0.568
		Combined	0.571	**0.628**
		Human	0.545	**0.613**
T1D1	T1D vs. C	Bacterial	**0.497**	0.465
		Combined	**0.624**	0.571
		Human	**0.758**	0.646
AL1	ALL vs. AML	Bacterial	**0.725**	0.688
		Combined	**0.755**	0.619

## Discussion

This work demonstrates a variety of computational proteomics methods utilizing the MetaProD pipeline that can aid precision medicine or other research. This includes highlighting species or genus level differences, identification of differentially expressed bacterial proteins in addition to human proteins when analyzing human diseases, and including bacterial proteins in overall analysis while illustrating some of the weaknesses.

Both the identification of species or genus of interest for a disease and identification of differentially expressed proteins for each phenotype can inform future studies. MS-based searches can focus on those proteomes or proteins in the search database rather than having to use the entire set of bacterial proteomes or pan-proteomes. This may significantly decrease search time and eliminate some issues with protein inference where including many species containing similar proteins in a FASTA protein database (especially those unlikely to be in the sample) may result in proteins being assigned to the incorrect species.

Predictive models can also be aided by the use of bacterial peptides or proteins in addition to human as shown by increases in mean AUC scores for some cases. The bacterial data tended to have significantly higher percents of missing values as previously shown. This meant that it had a higher percentage of data requiring imputation, which in this case was replacing the missing values with zeros. Imputation could have contributed to the decrease in bacterial scores compared to human. PEMM was used to impute missing values when identifying differentially expressed proteins but was not used during the ML models due to the potential of data leakage when using the entire data set for imputation. Specific methods for imputing MS data in ML models could help improve scores. A focus on identification during MS processing in the laboratory to avoid the need for imputation would also help avoid any biases that may be introduced when imputing missing data when the reason for the missing data is unclear.

Bacterial identification using mass spectrometry remains challenging and a variety of techniques exist not used in these studies that may improve identification of bacterial or differentially expressed proteins, such as using multiplexing reagents to label samples or using data-independent acquisition (DIA) when performing MS-analysis. The data sets in this study were label-free and analyzed using data-dependent acquisition (DDA) but one could expect improved results using other techniques.

DIA might have improved peptide identification across more samples and thus result in filtering fewer peptides compared to the DDA results here. DDA tends to lead to identification of more abundant proteins, which may be problematic when specifically looking for bacterial proteins in samples also containing human proteins. For these samples, the bacterial proteins may not be selected for fragmentation in the second step during MS/MS processing. Studies focusing specifically on identifying bacterial proteins can also aid identification as many studies focus only on human proteins and therefore experiments tend to be designed with this in mind.

More research and larger sample sizes are needed to fully evaluate possible improvements when using microbial proteins for host phenotype prediction. The results here suggest that with improved data gathering techniques, microbial proteins could offer improvements in AUC scores in ML classification while offering additional biomarkers for study and analysis. The use of microbial proteins for these purposes may need further validation.

## Data Availability

All MS data used is publicly available at the ProteomeXchange Consortium (http://www.proteomexchange.org) with identifiers PXD007819 (CD2), PXD008675 (CD3), PXD008870 (T1D1), and PXD011515 (AL1). MetaProD is available at https://github.com/mgtools/MetaProD. Peptide identification results and differentially abundant proteins are available at https://github.com/mgtools/MetaProD-ML. Machine Learning models including training and testing are available at the same repository as notebooks.
